# Nrf2 induces cisplatin resistance via suppressing the iron export related gene SLC40A1 in ovarian cancer cells

**DOI:** 10.18632/oncotarget.19548

**Published:** 2017-07-25

**Authors:** Jianfa Wu, Lingjie Bao, Zhenbo Zhang, Xiaofang Yi

**Affiliations:** ^1^ Department of Gynecology, Obstetrics and Gynecology Hospital, Fudan University, Shanghai, China; ^2^ Department of Obstetrics and Gynecology of Shanghai Medical School, Fudan University, Shanghai, China; ^3^ Shanghai Key Laboratory of Female Reproductive Endocrine Related Diseases, Shanghai, China; ^4^ Department of Obstetrics and Gynecology, Shanghai First People’s Hospital, Shanghai Jiaotong University, Shanghai, China

**Keywords:** Nrf2, SLC40A1, chemoresistance, ovarian cancer, iron

## Abstract

Induction of Nuclear factor erythroid 2 (NF-E2)-related factor 2 (Nrf2) has been demonstrated to be involved in cisplatin resistance in ovarian cancer. Solute carrier family 40 member 1 (SLC40A1) is an iron exporter, which possesses many putative Nrf2 binding sites. Here we hypothesize that it may be a possible downstream gene of Nrf2. Elevated level of Nrf2 and reduced level of SLC40A1 were found in cisplatin–resistant ovarian cancer cells as compared with cisplatin-sensitive ovarian cancer cells. Exogenous knockdown of Nrf2 leaded to increased expression of SLC40A1. While overexpression of Nrf2 resulted in decreased expression of SLC40A1. Chromatin immunoprecipitation (ChIP) and dual-luciferase reporter assay revealed that Nrf2 inhibited the transcription of SLC40A1. Overexpression of SLC40A1 was able to reverse cisplatin resistance induced by Nrf2, while knockdown of SLC40A1 restored cisplatin resistance and increased iron concentration. Desferal, an iron chelator, was found to overcome cisplatin resistance through iron deprivation. Its function was boosted when combined with brusatol, an Nrf2 inhibitor. Taken together, this study first demonstrated that Nrf2 could transcriptionally suppress the expression of SLC40A1. Iron overload induced by SLC40A1 resulted in cisplatin resistance in ovarian cancer. Targeting iron metabolism may be a new therapeutic strategy to reverse drug resistance in ovarian cancer treatment.

## INTRODUCTION

Ovarian cancer is one of the deadliest gynecological malignancies. Cytoreductive surgery and platinum-based chemotherapy are the first-line treatment [1, 2]. However, up to 80 % of these patients would experience multiple tumor relapse because of chemoresistance [3]. Chemoresistance has been a main obstacle for ovarian cancer treatment. Until now, the mechanism of chemoresistance in ovarian cancer remains unknown.

Nuclear factor erythroid 2 (NF-E2)-related factor 2 (Nrf2) is a key transcriptional factor involved in protecting cells against oxidative stresses. Nrf2 has been found to promote cell survival though activating many ARE-bearing genes, such as autophagy signaling associated genes [4, 5], ABC transporter family genes and cellular redox associated genes [6–8]. Moreover, constitutive expression of Nrf2 has been observed in many cancer cells [9–11]. In addition, our previous study reveals that Nrf2 contributes to cisplatin resistance in ovarian cancer [12]. However, the mechanism of cisplatin resistance induced by Nrf2 is still not fully understood.

Furthermore, abnormal iron metabolism is also associated with cancer development [13]. Persistent iron stimulation has been thought to be a high-risk factor for ovarian cancer formation [14]. Inversely, low dietary iron results in less rapid growth of tumor [15]. SLC40A1 is a novel iron metabolism associated gene, which is known as the only iron exporter gene [16]. The normal expression of SLC40A1 is essential to iron metabolism homeostasis. Moreover, many precious articles have revealed that abnormal iron metabolism induced by SLC40A1 mutation or intron sequence polymorphism is associated with autosomal dominant hemochromatosis, inflammatory reaction, and occurrence of liver cancer [17–22]. Our precious microarray data revealed that SLC40A1 was a potential downstream gene of Nrf2 [23]. Interestingly, further bioinformatics analysis indicated that there were many putative Nrf2 binding sites in the promotor of SLC40A1 (http://alggen.lsi.upc.es/cgi-bin/promo_v3/promo/promoinit.cgi?dirDB=TF_8.3). So we hypothesize that iron metabolism signaling pathway mediated by SLC40A1 is involved in cisplatin resistance induced by Nrf2.

In this study, we try to confirm Nrf2’s regulation on SLC40A1, and explore the role of iron metabolism in chemoresistance in ovarian cancer.

## RESULTS

### Expression of Nrf2 and SLC40A1 in different ovarian cancer cells

**Figure 1:**
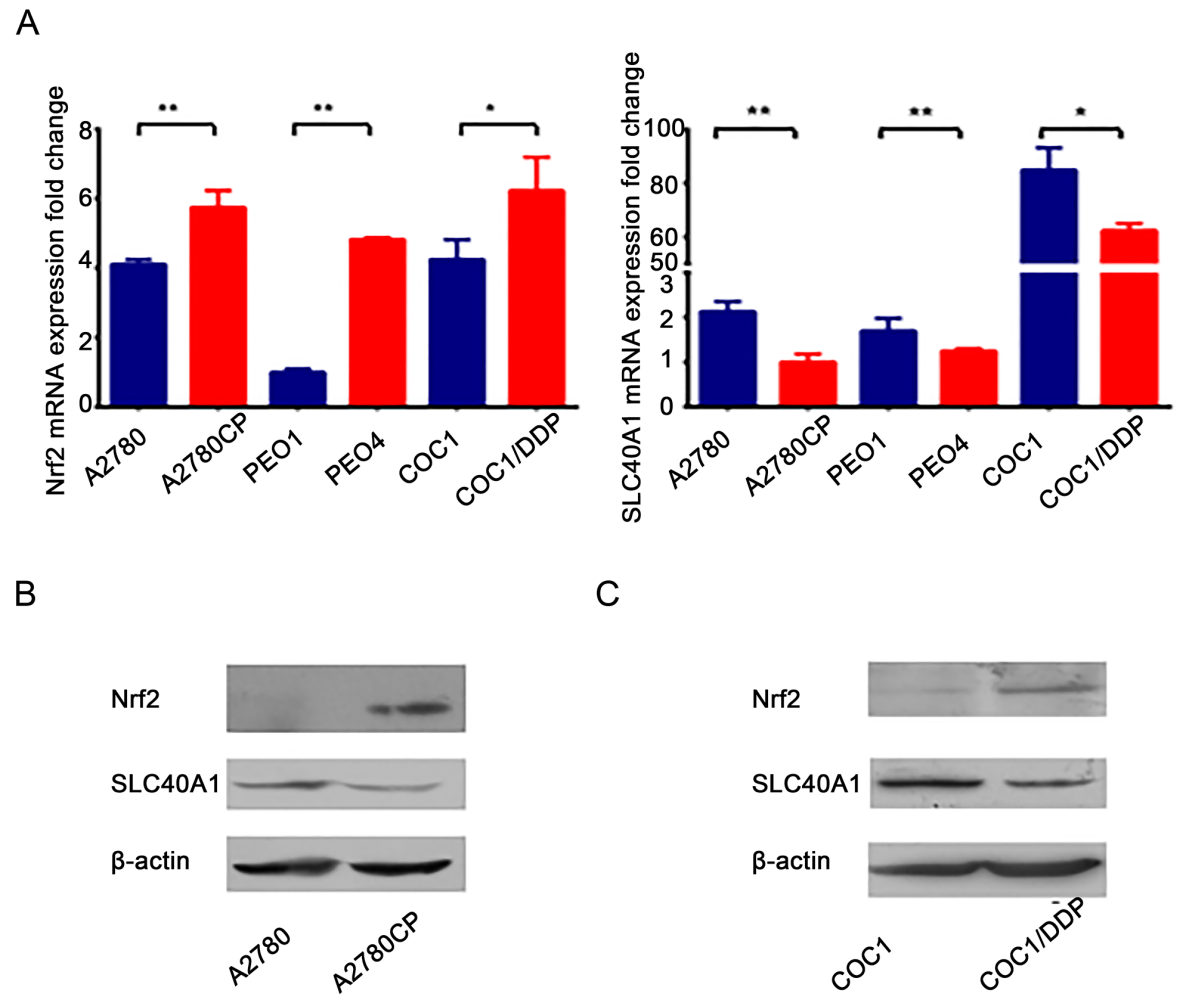
SLC40A1 and Nrf2 expression in cisplatin-sensitive ovarian cancer cells and cisplatin-resistant ovarian cancer cells **(A)** The mRNA expressions of Nrf2 and SLC40A1 in cisplatin-sensitive ovarian cancer cells (blue bar) and their derived cisplatin-resistant ovarian cancer cells (red bar) were determined by qRT-PCR. **(B)** Western blot was employed to detect the protein expression of Nrf2 and SLC40A1 in A2780 and its derived A2780CP ovarian cancer cells. **(C)** The protein expression of Nrf2 and SLC40A1 in COC1 and its derived COC1/DDP ovarian cancer cells were also determined through Western blot, ^*^ P<0.05, ^**^ P<0.01.

To study whether there is a relationship between Nrf2 and SLC40A1, we analyzed their mRNA expression in three cisplatin-sensitive ovarian cancer cells (A2780, COC1, PEO1) and their derived cisplatin-resistant ones (A2780CP, COC1/DDP, PEO4). Elevated mRNA level of Nrf2 and reduced mRNA level of SLC40A1 were found in three cisplatin–resistant ovarian cancer cells as compared with their corresponding cisplatin-sensitive ovarian cancer cells (Figure 1A). Moreover, A2780 and A2780CP, along with COC1 and COC1/DDP, which possessed relatively higher Nrf2 expression, were selected for the following experiments. Consistent with mRNA data, elevated protein level of Nrf2 and reduced protein level of SLC40A1 were also found in two cisplatin–resistant ovarian cancer cells (Figure 1B, 1C). This result suggests us that there may be an opposite interaction between Nrf2 and SLC40A1.

### Nrf2 inhibits the expression of SLC40A1

**Figure 2:**
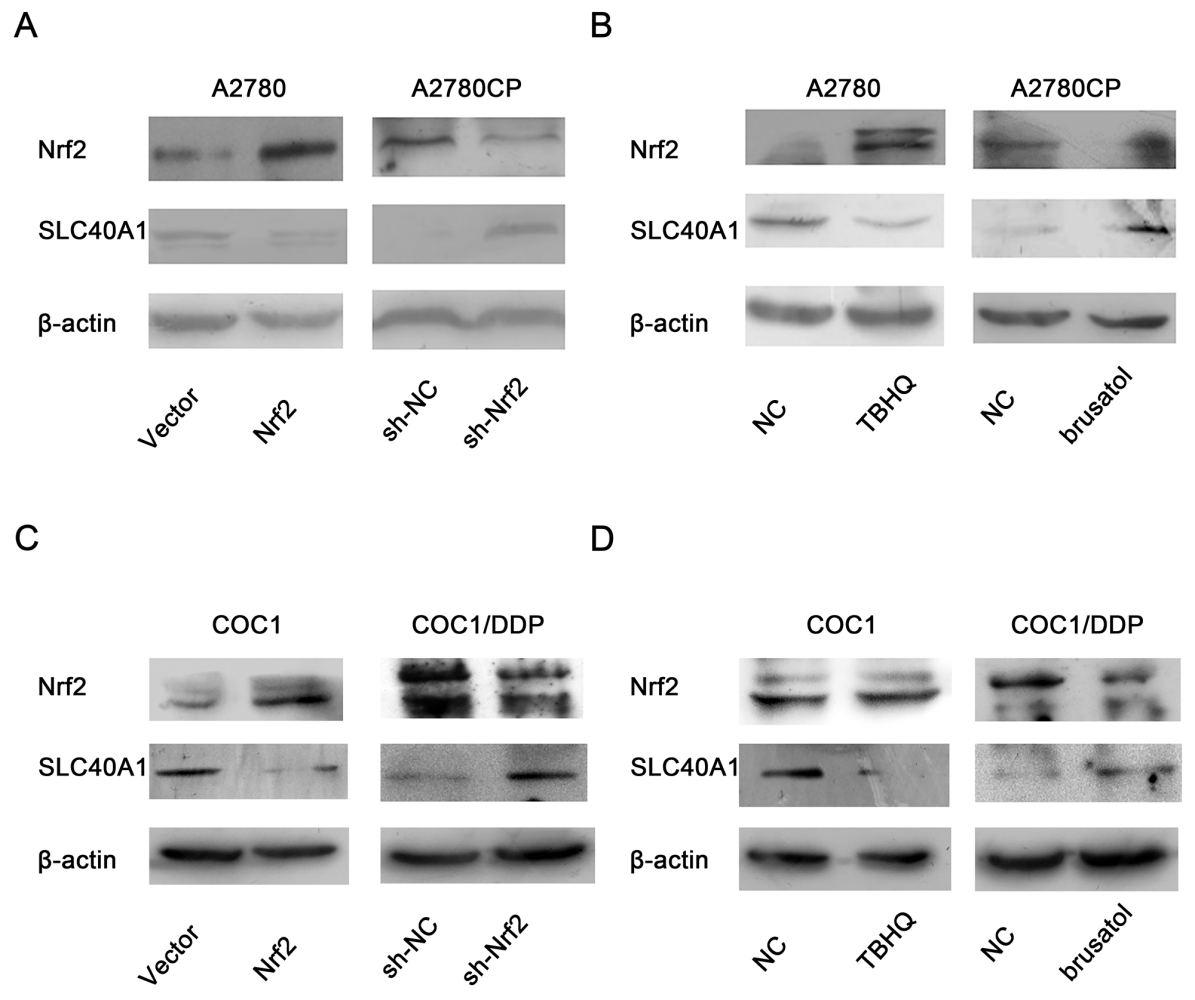
The inhibition of Nrf2 on SLC40A1 in ovarian cancer cells **(A)** The protein expressions of SLC40A1 were detected, when A2780 or A2780CP was treated with PCDH-Nrf2 vector or Nrf2 shRNA for 72h. **(B)** The protein expressions of SLC40A1 were determined, when A2780 was treated with Nrf2 activator (TBHQ, 50umol/l) for 16h, or A2780CP was treated with Nrf2 inhibitor (brusatol, 50nmol/l) for 3h. **(C)** The protein expressions of SLC40A1 were detected, when COC1 or COC1/DDP was treated with PCDH-Nrf2 vector or Nrf2 shRNA for 72h. **(D)** The protein expressions of SLC40A1 were determined, when COC1 was treated with Nrf2 activator (TBHQ, 50umol/l) for 16h, or COC1/DDP was treated with Nrf2 inhibitor (brusatol, 50nmol/l) for 3h.

Because bioinformatics analysis indicated that there are many putative Nrf2 binding sites in the promotor of SLC40A1, we hypothesize that SLC40A1 is one of down-stream genes of Nrf2, which is negatively regulated by the latter. To test this hypothesis, we adopted two methods to alter the expression of Nrf2 in order to observe the expression change of SLC40A1. Interestingly, it revealed that overexpression of Nrf2 by pCDH-CMV-MCS-EF1-copGFP-Nrf2 or tert-Butylhydroquinone (TBHQ) both inhibited the expression of SLC40A1 in A2780 (Figure 2A, 2B), while knockdown of Nrf2 by pLenR-shNrf2 or brusatol enhanced the expression of SLC40A1 in A2780CP (Figure 2A, 2B). Furthermore, another pair of ovarian cancer cell line was employed to test this result. Similar change of SLC40A1 was found in COC1 and COC1/DDP ovarian cancer cells (Figure 2C, 2D). This data suggests that SLC40A1 is a downstream gene of Nrf2 and Nrf2 can inhibit the expression of SLC40A1.

### SLC40A1 inverts cisplatin resistance induced by Nrf2

**Figure 3:**
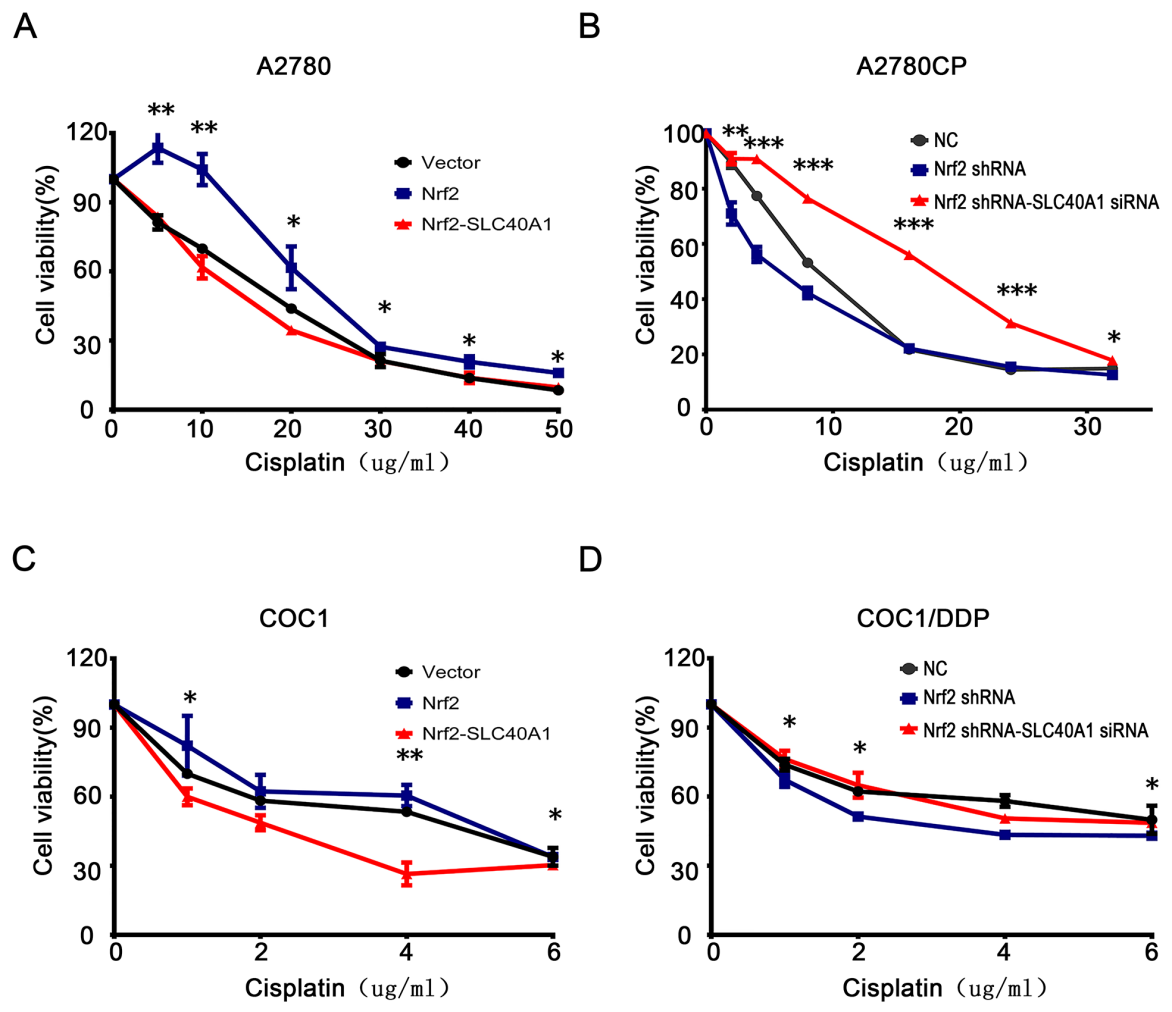
SLC40A1 inverts cisplatin resistance induced by Nrf2 **(A** and **C)** A2780 **(A)** or COC1 **(C)** was transfected with control vector (2ug), PCDH-Nrf2 plasmid (2ug), and PCDH/SLC40A1 (2ug) for 48 h. Then, they were treated with different levels of cisplatin for 24h to detect cell viability. **(B** and **D)** A2780CP **(B)** or COC1/DDP **(D)** was transfected with control siRNA (50nM), Nrf2 shRNA (2ug), and SLC40A1 siRNA (50nM) for 48 h. Then, they were treated with different levels of cisplatin for 24h to detect cell viability. ^*^ P<0.05, ^**^ P<0.01, ^***^ P<0.001.

To test whether SLC40A1 is a novel downstream gene of Nrf2, standard regression test was performed. The result revealed that co-transfected with SLC40A1 and Nrf2 plasmid significantly reduced cell viability compared to Nrf2 plasmid alone in A2780 and COC1 (Figure 3A, 3C). While co-transfected with SLC40A1 siRNA and Nrf2 shRNA showed higher cell viability compared to Nrf2 shRNA alone (Figure 3B, 3D). This suggests us that SLC40A1may be a downstream gene of Nrf2.

### Nrf2 transcriptionally suppresses the expression of SLC40A1

**Figure 4:**
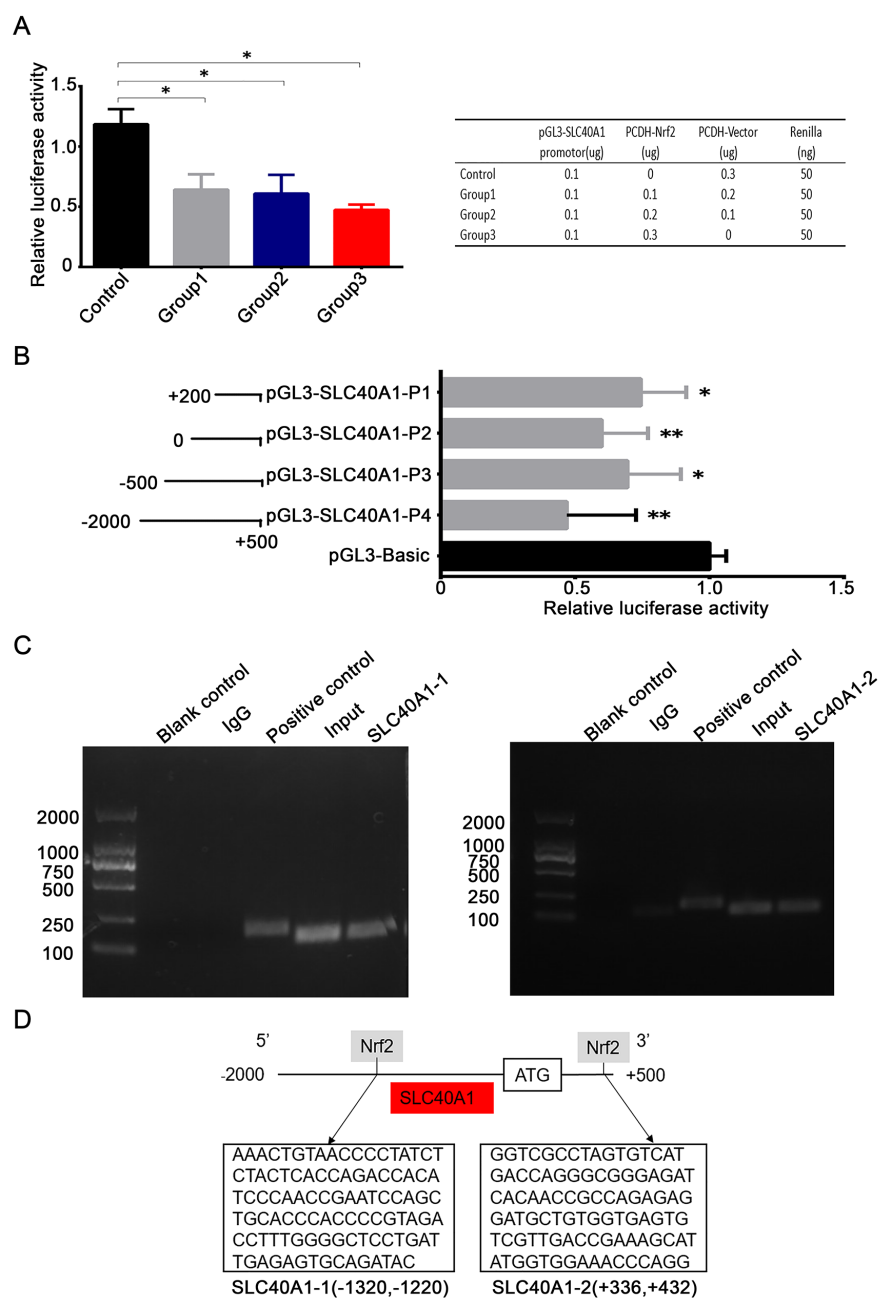
Nrf2 transcriptionally suppresses the expression of SLC40A1 **(A)** 5^*^10^3^ HEK-293T cells were transfected with different concentrations of PCDH-Nrf2 plasmid and pGL3-SLC40A1 reporter gene plasmid for 48 h. Then the promotor activity of SLC40A1 was determined by dual-luciferase reporter assay. **(B)** Left panel, different length of SLC40A1 promotor sequences were established according to putative binding sites and were linked to the reporter gene vector pGL3. Right panel, the relative luciferase activities among different SLC40A1 promotor sequences were analyzed by luciferase assay. **(C)** The interaction of Nrf2 with the SLC40A1 promotor was assayed by ChIP analysis. Numerous primers were designed according to the putative binding sites to detect the possible binding site. The reagent with no DNA was used as blank control. Total DNA was extracted and used as input. The normal mouse IgG was employed as the negative control. The GAPDH antibody was used as the positive control. **(D)** Sequence analysis indicated that there were two Nrf2 binding sites in the promotor of SLC40A1 (+336∼ +432, -1320∼ -1220). ^*^ P<0.05, ^**^ P<0.01.

To study the regulatory mechanism of Nrf2 to SLC40A1, pGL3-SLC40A1 promotor reporter gene plasmid was established. Relative luciferase activity analysis indicated that the transcription of SLC40A1 was significantly inhibited when pCDH-CMV-MCS-EF1-copGFP-Nrf2 plasmid was co-transfected (P<0.05). But no obvious dose-dependent relation was observed when it was given different quantity of pCDH-CMV-MCS-EF1-copGFP-Nrf2 plasmid (Figure 4A). This result further suggests us that Nrf2 can suppress the transcription of SLC40A1. Then, to search the binding site of Nrf2 within SLC40A1 promotor region. Different truncated bodies of SLC40A1 promotor sequence were designed and constructed. The activation region of SLC40A1 promotor was confirmed by comparison of relative luciferase activity of every truncated body. Interestingly, co-transfection of each truncated body and pCDH-CMV-MCS-EF1-copGFP-Nrf2 plasmid showed lower relative luciferase activity compared to pGL3-BASIC control (Figure 4B, P<0.05). This suggests us that there was one or more binding sites within SLC40A1 promotor region. Next, ChIP analysis was employed to test the transcriptional inhibition of Nrf2 to SLC40A1 *in vivo*. Through designing different primes aimed at the putative binding sites, it was found that there was an interaction between Nrf2 and SLC40A1 (Figure 4C). Furthermore, analysis of the synthesized DNA sequences indicated that Nrf2 interacted with SLC40A1 within two binding regions (+336∼+432,-1220∼-1320) (Figure 4D).

### The positive feedback reaction of SLC40A1 on Nrf2

**Figure 5:**
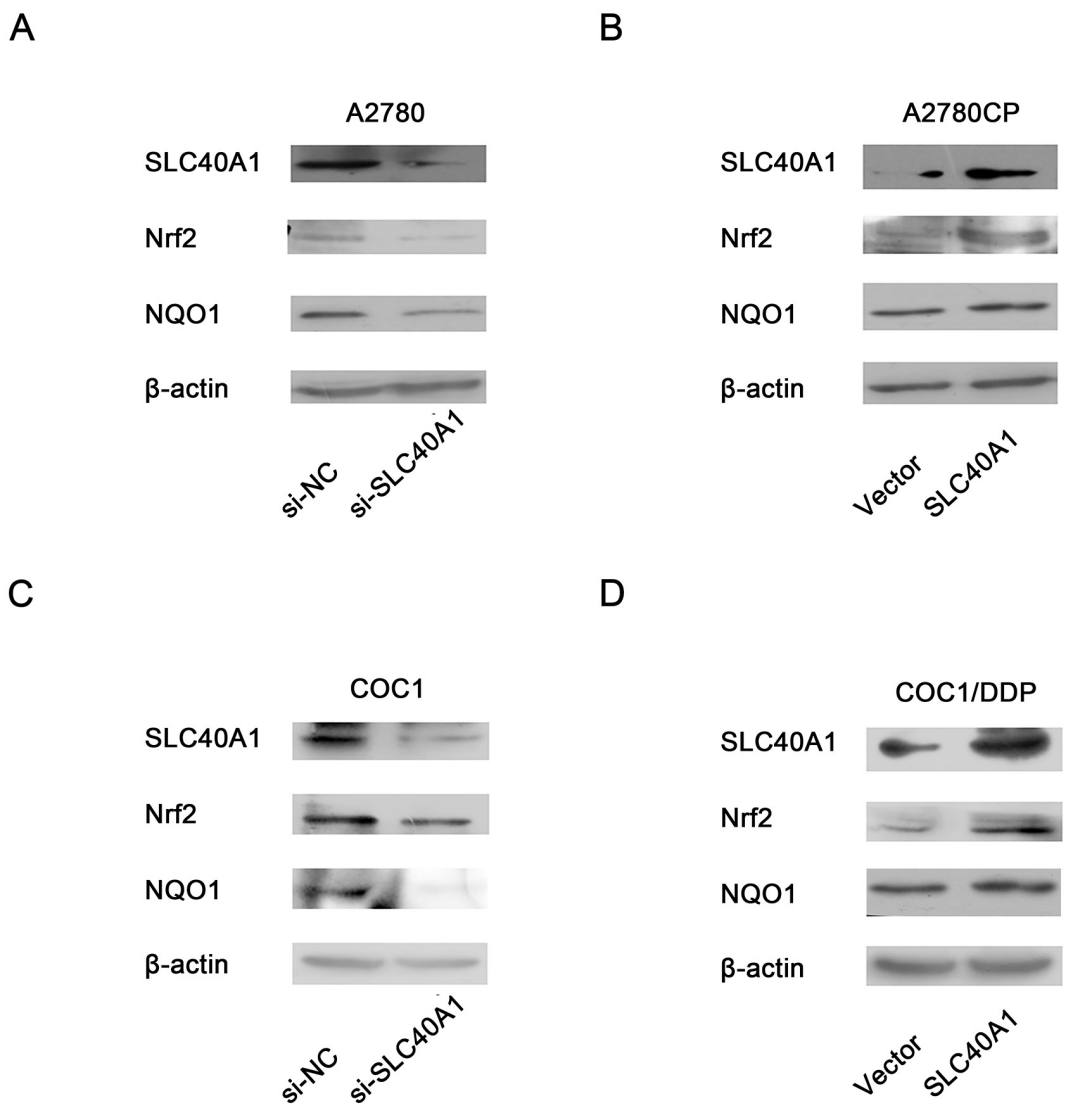
Positive feedback reaction of SLC40A1 on Nrf2 **(A** and **C)** Western blot was employed to determine the protein expression of Nrf2/NQO1, when SLC40A1 expression was inhibited with specific siRNA (50nM) in A2780 **(A)** and COC1 **(C)** ovarian cancer cells. **(B** and **D)** The protein expression of Nrf2/NQO1were detected by western blot, when the expression of SLC40A1 was enhanced through PCDH-SLC40A1 transfection in A2780CP **(B)** and COC1/DDP **(D)** ovarian cancer cells.

To figure out whether there was any feedback reaction of SLC40A1 on Nrf2, the protein level of Nrf2 was determined by western blot after SLC40A1 was upregulated or knockdown. Interestingly, when SLC40A1 was knockdown in A2780, the expression of Nrf2 and its downstream gene NQO1 decreased (Figure 5A). Inversely, upregulation of SLC40A1 in A2780CP resulted in increased Nrf2 and NQO1 protein expression (Figure 5B). To test this result, we employed another pair of ovarian cancer cells to repeat this experiment. Similarly, it was found that SLC40A1 promoted the expression of Nrf2 and NQO1 in COC1 and COC1/DDP (Figure 5C, 5D). These results suggest that there is an interaction loop between Nrf2 and SLC40A1, but not a simple linear relationship.

### SLC40A1 regulates iron metabolism to overcome cisplatin resistance

**Figure 6:**
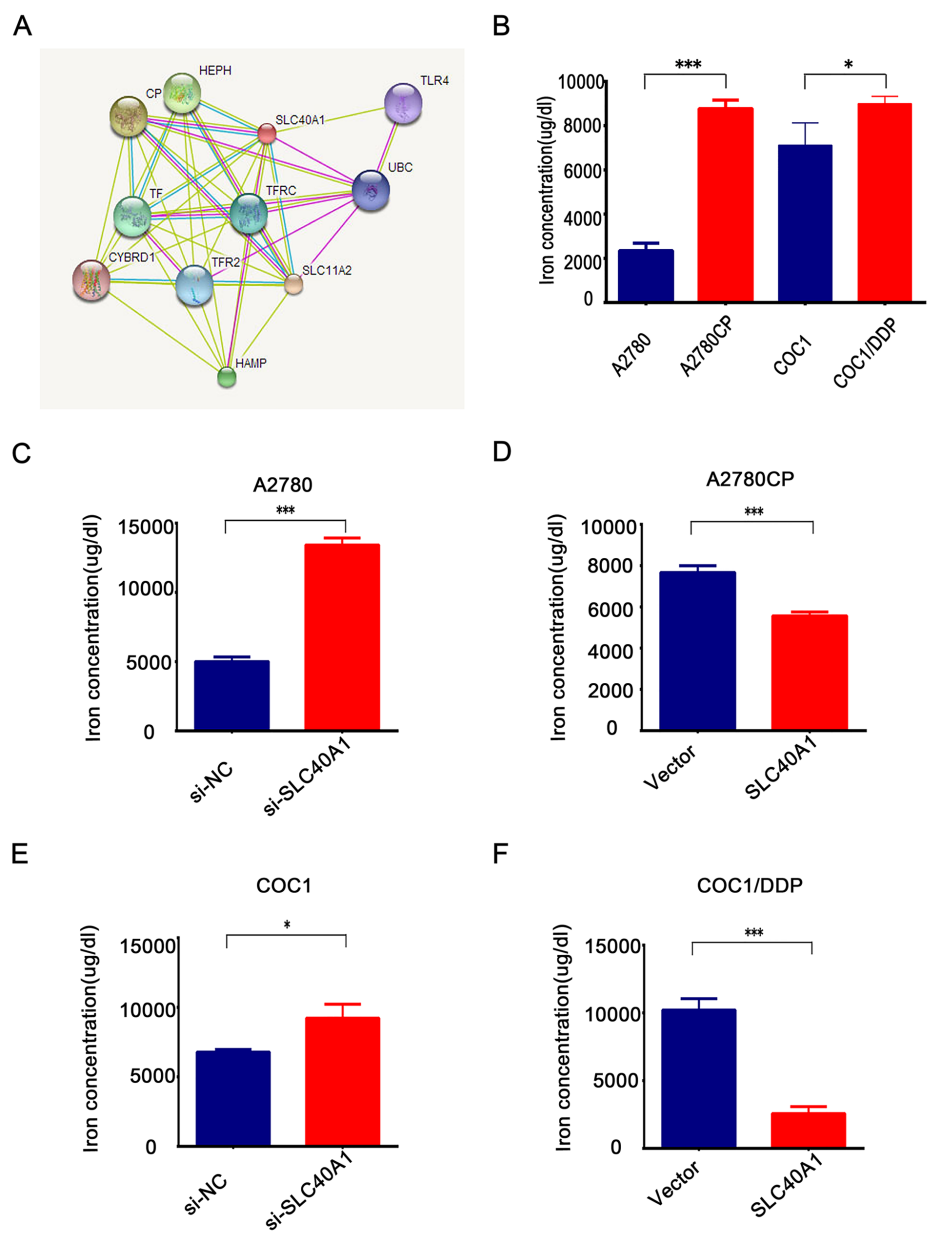
SLC40A1 regulates iron metabolism to induce cisplatin resistance in ovarian cancer **(A)** Bioinformatics analysis indicated that most interact proteins of SLC40A1 are involved in iron metabolism. **(B)** The concentration of iron in cisplatin-sensitive ovarian cancer cells (blue bar) and their corresponding cisplatin-resistant ovarian cancer cells (red bar) were determined by QuantiChrom™ Iron Assay Kit. **(C** and **E)** After transfecting with SLC40A1 siRNA (50nM) or its specific control for 72 h, 2^*^10^5^ A2780 or COC1 ovarian cancer cells were employed to detect intracellular iron concentration by QuantiChrom™ Iron Assay Kit. **(D** and **F)** After overexpression of SLC40A1 with PCDH-SLC40A1(2ug) or its control vector for 72 h, intracellular iron concentration was determined in A2780CP or COC1/DDP by QuantiChrom™ Iron Assay Kit. ^*^ P<0.05, ^***^ P<0.001.

Previous studies have demonstrated that SLC40A1 was the only known iron exporter [24, 25]. The most discovered interact proteins of SLC40A1 are involved in iron metabolism (http://string-db.org/) (Figure 6A). To test the relation between iron metabolism and cisplatin resistance, two pairs of ovarian cancer cells with different cisplatin response were employed to detect the intracellular iron concentration. Interestingly, it was found that there were higher concentrations of iron in cisplatin-resistant cancer cells than cisplatin-responsive ovarian cancer cells (Figure 6B). This suggests us that iron overload in ovarian cancer cells is associated with cisplatin resistance. Moreover, it was revealed that intracellular iron concentration increased when SLC40A1 was knockdown by siRNA in A2780 (Figure 6C). However, when SLC40A1 was upregulated, intracellular iron reduced in A2780CP (Figure 6D). Similarly, it was confirmed that SLC40A1 was associated with iron export in COC1 and COC1/DDP (Figure 6E, 6F). This data suggests that SLC40A1 is an iron exporter gene and iron metabolism regulation may be a novel mechanism for cisplatin resistance in ovarian cancer.

### Desferal overcomes cisplatin resistance via reducing intracellular iron

**Figure 7:**
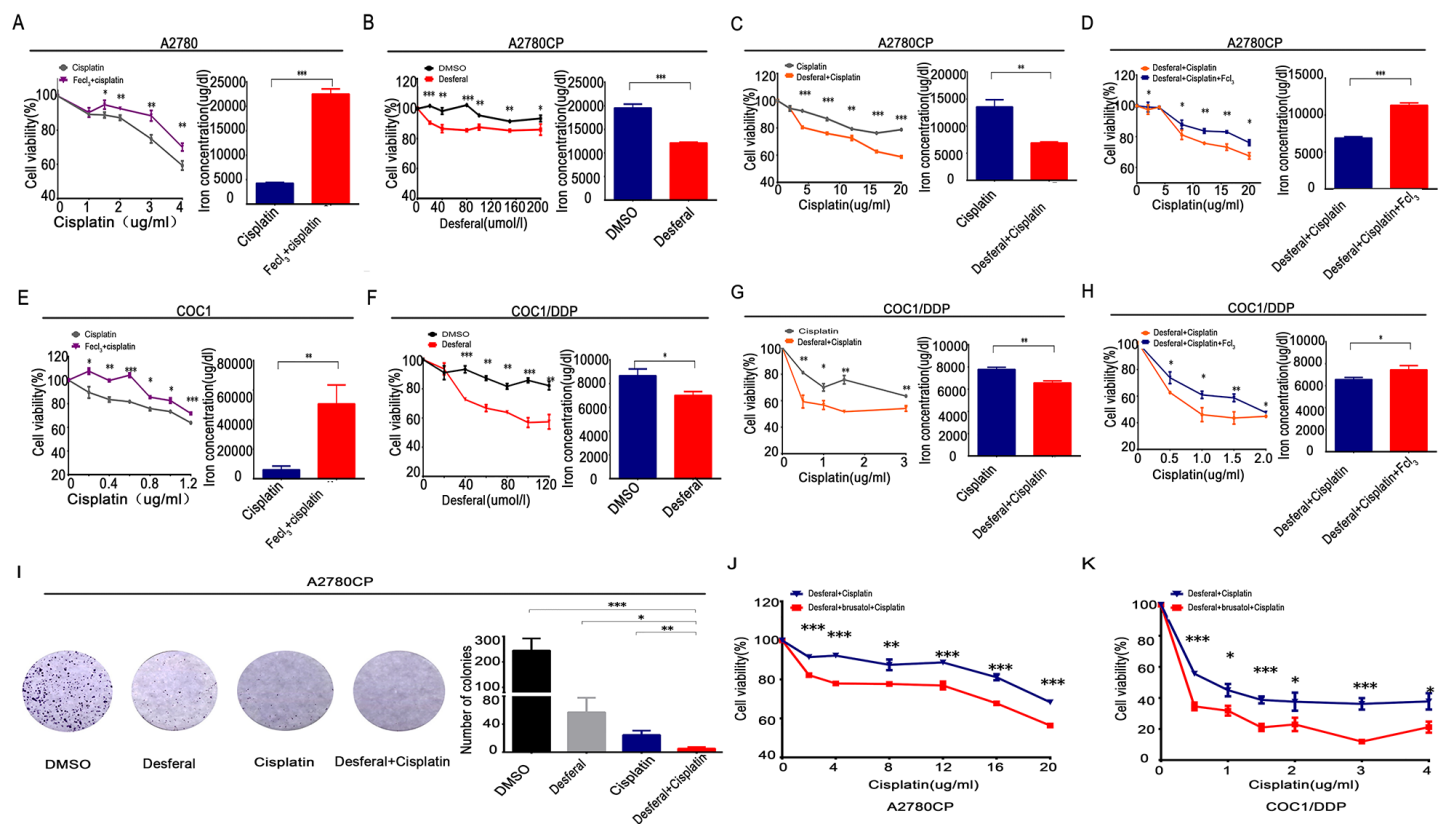
Desferal and brusatol inverts iron overload to sensitize ovarian cancer cells to cisplatin **(A** and **E)** After treatment with Fecl_3_ (20umol/l) and different concentrations of cisplatin for 24 h, QuantiChrom™ Iron Assay Kit was employed to detect the intracellular iron concentration, and CCK8 assay was used to determine the cell viability in A2780 **(A)** and COC1 **(E)**. **(B** and **F)** After treatment with different concentrations of desferal for 24 h, QuantiChrom™ Iron Assay Kit was employed to detect the intracellular iron concentration, and CCK8 assay was used to determine the cell viability in A2780CP **(B)** and COC1/DDP **(F)**. **(C** and **G)** Intracellular iron concentration and cell viability were determined as above, when A2780CP **(C)** and COC1/DDP **(G)** were treated with desferal (80umol/l) and different concentrations of cisplatin for 24 h. **(D** and **H)** According to the proportion of 1 to 1, Fecl_3_ (80umol/l) was added to combine the Fe^3+^ binding sites on the desferal. Then, A2780CP **(D)** and COC1/DDP **(H)** were treated with different concentrations of cisplatin for 24 h, and intracellular iron concentration and cell viability were determined as above. **(I)** 2^*^10^3^ A2780CP ovarian cancer cells were treated with DMSO, desferal (20umol/l), cisplatin (10ug/ml), or desferal (20umol/l) plus cisplatin (10ug/ml) for 24h. After 12 days, colony formation in cisplatin plus desferal group was lowest in all groups (P<0.05). **(J** and **K)** After treatment with desferal (80umol/l) plus brusatol (50nmol/l) for 24 h, cell viability assay indicated that cisplatin toxicity was significantly enhanced in A2780CP **(J)** and COC1/DDP **(K)**. ^*^ P<0.05, ^**^ P<0.01, ^***^ P<0.001.

**Figure 8:**
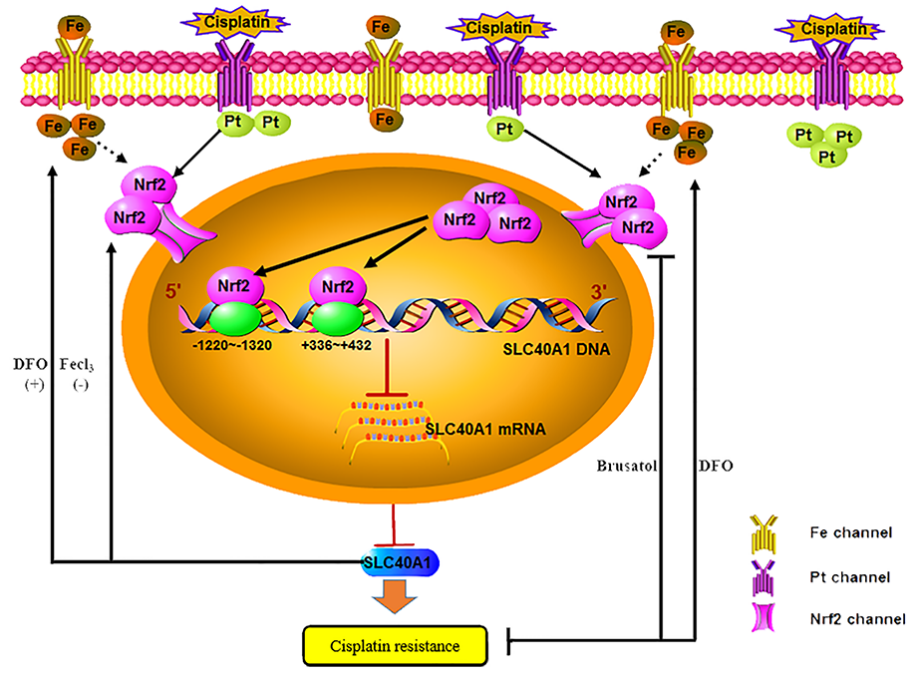
Schematic: SLC40A1 promotes iron export and prevents Nrf2 dependent cisplatin resistance in ovarian cancer Nrf2 is activated and transported into cell nucleus after cisplatin treatment in cisplatin-resistant ovarian cancer cells. Increased Nrf2 in cell nucleus combines to the binding sites (+336∼+432,-1220∼-1320) to transcriptionally inhibit the expression of SLC40A1, thus resulting in cisplatin resistance. However, SLC40A1 also has a positive feedback interaction with Nrf2. Moreover, as a novel iron exporter, abnormal expression of SLC40A1 is associated with cisplatin resistance and increased intracellular iron. While increased intracellular iron induced by Fecl3 results in cisplatin resistance. Inversely, desferal, an iron chelator, overcomes cisplatin resistance by reducing intracellular free iron. Brusatol enhances the antitumor effect of desferal by inhibiting expression of Nrf2 in cisplatin-resistant ovarian cancers.

Iron is an essential element involved in physiological activity. In this study, it was revealed that treatment with Fecl_3_ leadedto increased intracellular iron and resulted in cisplatin resistance in A2780 and COC1 (Figure 7A, 7E). Desferal, as an iron chelator, has been used as an effective reagent in clinic treatment for iron overload [26]. In this study, it was found that desferal alone could significantly reduce intracellular iron and cell viability in A2780CP and COC1/DDP (Figure 7B, 7F). Desferal accompanied with cisplatin also enhanced cisplatin toxicity in A2780CP and COC1/DDP (Figure 7C, 7G). Moreover, colony formation analysis revealed that desferal accompanied with cisplatin could better inhibit the colony formation (Figure 8A). However, given similar proportion of Fecl_3_ to combine the Fe^3+^ binding sites on the desferal, the intracellular iron and cell viability increased (Figure 7D, 7H). This data suggests that iron overload results in cisplatin resistance and Desferal overcomes cisplatin resistance through reducing intracellular iron in ovarian cancer.

### Brusatol enhances the antitumor effect of desferal to cisplatin-resistant ovarian cancers

It was revealed that enhanced expression of SLC40A1 sensitized ovarian cancer to cisplatin and also resulted in increased expression of Nrf2. However, increased Nrf2 expression contributed to cisplatin resistance. To obtain better chemotherapeutic effect, brusatol, an Nrf2 inhibitor, accompanied with desferal were adopted to assess their antitumor effect. Finally, we revealed that brusatol could enhance the antitumor effect of desferal (Figure 8B, 8C). This result suggests that brusatol combined with desferal can better overcome cisplatin resistance in ovarian cancer.

## DISCUSSION

Abnormal activation of Nrf2 antioxidant signaling pathway has been regarded as a crucial mechanism for chemoresistance in recent years [10, 27, 28], and numerous studies have attempted to explain the mechanism of chemoresistance induced by Nrf2 [29–31]. Until now, there is still limited knowledge about its mechanism.

SLC40A1, has been found to be associated with cisplatin resistance in ovarian cancer. Moreover, in this study it showed that Nrf2 could combine to the promotor of SLC40A1 and transcriptionally suppressed the expression of SLC40A1 in ovarian cancer. This provides us new understanding to cisplatin resistance induced by Nrf2. However, there have been still controversial about the interaction between Nrf2 and SLC40A1 at present. Some recent studies revealed that there was positive correction between Nrf2 and SLC40A1 in murine bone marrow-derived macrophages [21, 32]. So the interaction between Nrf2 and SLC40A1 is a complex physiological procedure. Perhaps there are different regulations among different binding sites, organs and species.

In this study, we also found that SLC40A1 could promote the expression of Nrf2 in return. This results tell us that there is an interaction loop between Nrf2 and SLC40A1, but not a simple linear relationship. To keep the iron homeostasis, SLC40A1 positively regulates the expression of Nrf2 to maintain its stability. But as a membrane protein, how SLC40A1 regulates Nrf2 is still unknown. To answer this question, we analyzed the interactive protein of SLC40A1 by online software (http://string-db.org/). Interestingly, most of the interactive proteins were found to be associated with iron metabolism. So we suppose that abnormal iron metabolism induced by SLC40A1 may be a potential reason for overexpression of Nrf2 associated genes. However, this hypothesis still needs further experiments to confirm in the future.

Precious studies have revealed that abnormal iron metabolism is closely related to the poor prognosis of tumor [33–36]. Moreover, desferal, as an iron chelator, has been found to sensitize cervical cancer to oxaliplatin through hCtr1 and TfR1 [37]. However, there has been no publications about iron overload and cisplatin resistance in ovarian cancer. In this study, it revealed that iron overload induced by SLC40A1 resulted in cisplatin resistance. While iron deprivation by desferal sensitized ovarian cancer cells to cisplatin. These results suggest that iron overload is associated with cisplatin resistance in ovarian cancer. Targeting iron metabolism may be a new method for ovarian cancer treatment. This finding first sheds new light on the knowledge of cisplatin resistance and iron metabolism.

Brusatol is an inhibitor of Nrf2, which directs Nrf2 to E3 ubiquitin ligase to reduce the activate Nrf2 level [38]. It has been found to inhibit Nrf2 and ameliorate chemoresistance both *in vivo* and *in vitro* in many cancers [39, 40]. In this study, lower expression of SLC40A1 and higher expression of Nrf2 was found to be associated with cisplatin resistance. Moreover, it was revealed that there had been a positive reaction of SLC40A1 on Nrf2. To obtain a better antitumor effect, simultaneously targeting SLC40A1 and Nrf2 was performed to assess its antitumor effect. Finally, it revealed that brusatol accompanied with desferal could significantly sensitize ovarian cancer to cisplatin than desferal alone. However, this result came from *in vitro* experiments, its risk/benefit ratio still needs *in vivo* studies and clinical trials to verify.

In summary, our results demonstrated that Nrf2 could transcriptionally inhibited the expression of SLC40A1 to mediate cisplatin resistance, which made us have a further understanding to cisplatin resistance mediated by Nrf2. Moreover, it was first revealed that iron overload resulted in cisplatin resistance in ovarian cancer. And desferal, as an iron chelator, can be used as an adjuvant agent to sensitize ovarian cancers to cisplatin through ameliorating iron overload. Furthermore, brusatol associated with desferal was found to be able to enhance the antitumor effect of desferal. This work deepens the understanding of cisplatin resistance in ovarian cancer, which opens a new chapter for study of chemoresistance in ovarian cancer. However, there is still a long way to go from *in vitro* experiments to clinical bedside. Future studies will focus on the mechanisms between iron metabolism and chemoresistance *in vivo*.

## MATERIALS AND METHODS

### Reagents

Desferal was purchased from Novartis Pharma (Schweiz AG, Swizerland). Cisplatin and tert-Butylhydroquinone (TBHQ), an activator of Nrf2 [41], were obtained from Aladdin (Aladdin industrial, Fengxian, Shanghai, China). Brusatol, an inhibitor of Nrf2 [29], was purchased from Rongbai (Shanghai, China). Fecl_3_ was obtained from Sinopharm Chemical Reagent Co. Ltd (Shanghai, China). The SYBR premix ExTaq II system was purchased from Takara (Otsu, Japan). Small interfering RNA of SLC40A1 and its nonspecific control were purchased from Riobobio (Guangzhou, China), and the siRNA sequences were shown in Table 1. pCDH-CMV-MCS-EF1-copGFP-Nrf2 plasmid, pLenR-shNrf2 plasmid and their specific control plasmid were purchased from Ivabio (Shanghai, China). Renilla plasmid was kindly provided by Pro. Ming Yao (State Key Laboratory of Oncogenes and Related Genes, Shanghai Cancer Institute, Renji Hospital, Shanghai Jiao Tong University School of Medicine, Shanghai, China). DMSO was purchased from Sigma (St. Louis, MO), which was used as solvent for desferal, TBHQ, Fecl3.

**Table 1:** SLC40A1 siRNA sequences

	Sense	antisense
#1	GGAUGGGUCUCCUACUACATT	UGUAGUAGGAGACCCAUCCAT
#2	GGAUUGACCAGUUAACCAATT	UUGGUUAACUGGUCAAUCCTT
#3	GGCUUGCUCGUAUUGAUUUTT	AAAUCAAUACGAGCAAGCCAA

### Cell culture

Human ovarian carcinoma cell line A2780 and its derived cisplatin-resistant cell line A2780CP, PEO1 and its derived cisplatin-resistant cell line PEO4 ovarian cancer were cultured as following. Cells were maintained in Dulbecco’s modified Eagle’s medium (DMEM) with 10% fetal bovine serum (Biowest, France) and penicillin (100 U/mL)/streptomycin (100 μg/mL), which were obtained as preciously described [12]. Ovarian carcinoma cell line COC1 and its resistant cell line COC1/DDP were purchased from Peking Union Medical College (Beijing, China), which were maintained in RPMI-1640 medium. While human epithelial kidney 293T cells (HEK293T), which was purchased from ATCC, was also grown in DMEM. All cells were cultured in a humidified 5% CO2 atmosphere at 37°C.

### Construction of SLC40A1 overexpression plasmid and SLC40A1 reporter gene plasmid

SLC40A1 CDS fragment was amplified with primer sequence as follows: Forward, AGAAGATTCTAGAGCTAGCGAATTCATGACCAGGGCGGGAGATCACAA and reverse, GCAGATCCTTCGCGGCCGCGGATCCTCAAACAACAGATGTATTTGCTTGA. The purified PCR products were cloned into the pCDH-CMV-MCS-EF1-puro plasmid. While, the luciferase reporter plasmid containing the SLC40A1 promotor was obtained from Genewiz (Suzhou, China), which was used as a template to amplify different length of truncated bodies of SLC40A1 promoter. The primers used were shown in Table 2. The purified different length of truncated bodies were connected to pGL3-Basic plasmid with Peasy-Uni Seamless Cloning and Assembly Kit according to the manufacturer’s recommendations (TRANSGEN, Beijing, China). Sequencing analyses were applied to confirm the integrity of all plasmid constructs.

**Table 2:** Primers for truncated bodies of SLC40A1 promoter

sense		antisense
#1	CTATCGATAGGTACCGCTATGGTTCACAGCAGAGC	ATCGCAGATCTCGAGCCAAAGTCGTCGTTGTAGTCT
#2	CTATCGATAGGTACCGCTATGGTTCACAGCAGAGC	ATCGCAGATCTCGAGTAAGAGCTGGGCCCGG
#3	CTATCGATAGGTACCGCTATGGTTCACAGCAGAGC	ATCGCAGATCTCGAGTACCTGTATTAAAAATTATTTTCG
#4	CTATCGATAGGTACCGCTATGGTTCACAGCAGAGC	ATCGCAGATCTCGAGTTTAGTTCACTCATCTGAGTATAC
#5	CTATCGATAGGTACCGCTATGGTTCACAGCAGAGC	ATCGCAGATCTCGAGGGATTTAAGATTCCCCTTCCACAG

### Transient transfection of the cell lines

Cells were seeded at 2^*^10^5^ Cells per well in 6-well plate. After 18–24 h, cells were transiently transfected using Lipofectamine 2000 according to the manufacturer’s recommendations (Invitrogen, Carlsbad, CA, USA) with 2ug plasmid DNA or 5ul siRNA. The transfection efficiency was determined by Quantitative real-time polymerase chain reaction (qRT-PCR) and western blot.

### Quantitative real-time polymerase chain reaction (qRT-PCR)

The TRIzol reagent (Invitrogen, Carlsbad, CA) was used for the extraction of total RNA from ovarian cancer cells. RT reactions were performed by RT kit according to the manufacturer’s recommendations (Takara, Japan). Then, The SYBR premix ExTaq II system was used to perform PCR analysis on the PRISM 7500 (ABI, USA). All qRT-PCR primers involved in this study were listed in Table 3. Every analysis was performed in three independent experiments and the levels of gene expression were determined by adopting 2^–ΔΔCt^ value.

**Table 3:** Primer sequences for different genes

	sense	antisense
Nrf2	ACACGGTCCACAGCTCATC	TGTCAATCAAATCCATGTCCTG
NQO1	ATGTATGACAAAGGACCCTTCC	TCCCTTGCAGAGAGTACATGG
HO-1	ACTGCGTTCCTGCTCAACAT	GGGCAGAATCTTGCACTTTGTT
SLC40A1	AACAAGCACCTCAGCGAGAG	CACATCCGATCTCCCCAAGT
β-actin	CTCCATCCTGGCCTCGCTGT	GCTGTCACCTTCACCGTTCC

### Western blot

Western blot was performed as preciously described [12]. Twenty-thirty microgram of different proteins were employed to perform western blot analysis. Specific Anti-SLC40A1 (1:300, Novus, USA), Anti-Nrf2 (1:500, Santa cruz, USA), Anti-NQO1 (1:500, Santa cruz, USA), Anti-Nrf2 (1:1000, Abcam, England), and β-actin (1:5000, Sigma, St. Louis, MO) were employed to determine their corresponding protein in ovarian cancer cells. Purified anti-rabbit IgG antibody and anti-mouse IgG antibody (1:5000, Cell Signaling Technology, Boston, USA) were chosen as the secondary antibody. SuperSignal West Femto Maximum Sensitivity Substrate (Themoscientific, Japan) was purchased to analyze the relative protein level. All blots were performed in triplicate.

### Cell viability assay

Cell viability was determined by Cell Counting Kit 8 (CCK8) assay according to the manufacturer’s recommendations (Dojindo, Japan). Briefly, 1× 10^4^ different ovarian cancer cells were seeded in 96-well plates and treated with different concentrations of compounds (desferal, Brusatol, Cisplatin or Fecl3) for 24-48h. Then, the CCK8 reagent was added to the medium and incubated for 2h for absorbance measurement with the Bio-Rad microplate reader at the wavelength of 450 nm (Hercules, CA, USA). All treatments were performed in triplicate.

### Dual-luceferase reporter assay

HEK293-T cells were seeded at a density of 5^*^10^3^/well in 96-well plate. After 24h, co-transfection was performed with pGL3-SLC40A1, pCDH-CMV-MCS-EF1-copGFP-Nrf2, pCDH-CMV-MCS-EF1-copGFP and Renilla plasmid. All plasmids were gently added to each well with Lipofectamine 2000 (Invitrogen, MA, USA). After 48h transfection, all cells were rinsed thoroughly by PBS. Then, 20ul cell lysis buffer was added to each well using the Dual Luciferase Assay System (Promega, Wisconsin, USA) according to the manufacturer’s recommendations. When all cells were splitted thoroughly, 50ul firefly luciferase reagent was added to each well. Firefly luciferase levels were analyzed with PerkinElmer 2030 Multilabel Reader (PerkinElmer). Finally, 50ul renilla luciferase reagent was added into each well, and renilla luciferase levels were analyzed. The relative luciferase activity of each sample was obtained with dividing firefly luciferase by renilla luciferase. All treatments were performed in triplicate.

### Chromatin immunoprecipitation (ChIP) assay

ChIP assays were performed according the EZ-ChIP™ Kit (17-371, Millipore, Darmstadt, Germany). When cell confluence of A2780CP reached 80%, 30ug/ml cisplatin was added to culture medium to promote protein and chromatin crosslinking. After 4 hours. 1% formaldehyde was given to each 10 cm culture plate to fix the protein-chromatin complex for 10 min and the residual formaldehyde was neutralized with glycine for 5 min at room temperature. The following process of ChIP was performed as previously described [13]. Soluble chromatin was immunoprecipitated using the Nrf2 antibody (Abcam, 1:50) against DNA binding protein. Then, different specific primers were designed according to the putative binding sites (Table 4). Real-time PCR was performed using these specific primers.

**Table 4:** Primers for ChIP assay

	sense	antisense
#1	GTATCTGCACTCTCAATCAGGAGC	AAACTGTAACCCCTATCTCTACTCAC
#2	CCTGGGTTTCCACCATATGCTTTC	AGTTCCTTGCACTCCTGTTAACAA
#3	CCTGGGTTTCCACCATATGCTTTC	GGTCGCCTAGTGTCATGACCA
#4	GACCACATCCCAACCGAATC	CACAGGCCAGACTGACACCC
#5	GACCACATCCCAACCGAATC	CTGACACCCAGTAGTGAAGG
#6	GATAGCAGCCGCAGAAGAGC	CCACCTGGGTTTCCACCATA

### Iron concentration assay

Cells were seeded at 2^*^10^5^ Cells per well in 6-well plate. Treatment with different reagents for 24-72h, cells were washed and splitted as preciously described [42]. Iron concentration assay was determined by the QuantiChromTM Iron Assay Kit (DIFE-250, BioAssays Systems, CA, USA) according to the manufacturer.

### Statistics

All statistical analyses were performed using SPSS software (version 18.0). Two independent samples student t-test was used to compare differences between two groups. The differences were considered significant when a two-sided P value < 0.05.

## Abbreviations

SLC40A1: Solute carrier family 40 member 1
Nrf2: Nuclear factor erythroid 2 (NF-E2)-related factor 2
ABC: ATP-binding cassette
TBHQ: tert-Butylhydroquinone
siRNA: Small interfering RNA
qRT-PCR: Quantitative real time polymerase chain reaction
DMEM: Dulbecco’s modified Eagle’s medium
ChIP: Chromatin immunoprecipitation
NQO1: NAD(P)H quinone dehydrogenase 1
c-Myc: v-myc avian myelocytomatosis viral oncogene homolog
NF-κB: nuclear factor kappa B
TfR1: transferrin receptor
hCtr1: hypocretin receptor 1
CCK8: Cell Counting Kit 8
